# Laser tag training reduces knee abduction moments and improves performance during change-of-direction movements

**DOI:** 10.3389/fspor.2026.1686129

**Published:** 2026-02-23

**Authors:** Maurice Mohr, Fabio De Santis

**Affiliations:** Department of Sport Science, University of Innsbruck, Innsbruck, Austria

**Keywords:** ACL rupture, anterior cruciate ligament injury, injury prevention, joint biomechanics, side-cutting, sidestepping, training gamification

## Abstract

**Introduction:**

A high peak knee abduction moment (pKAM) during change-of-direction (COD) movements is considered a risk factor for non-contact injury to the anterior cruciate ligament during multidirectional team sports. COD technique training aimed at avoiding injury-prone movement patterns can lead to reductions in the pKAM but may limit COD performance. In this study we investigated a novel constraints-led training approach based on a 1-on-1 laser tag game, which may optimize COD movement patterns simply through the game's constraints rather than technique instructions. Specifically, we investigated whether the constraints-led training approach reduces the pKAM but with COD performance maintained or even improved.

**Methods:**

Twelve highly active individuals (75% female) with experience in COD sports completed an eight-week laser tag training (LASG) intervention while COD biomechanics and performance were obtained from 3D motion capture and full-body musculoskeletal modeling of a maximum-speed 135° COD. Training effects in the LASG group were compared to training effects of traditional COD technique training (CODG) and linear sprint training (CG) obtained from a previous study.

**Results:**

After the training, the LASG showed a statistically significant reduction in the pKAM compared to pre-training (*p* = 0.038, Cohen's d = 0.63) with magnitudes comparable to the CODG (*p* = 0.056, d = 0.58). Further, the LASG showed improvements in COD performance, quantified through statistically significant reductions in COD completion times (*p* < 0.001, d = 2.47), which was not observed for CODG (*p* = 0.898, d = 0.04).

**Discussion:**

In conclusion, an eight-week laser tag training intervention can reduce the pKAM and improve performance during a maximum-speed COD and thus may be a useful tool in ACL injury prevention training.

## 1 Introduction

Non-contact injuries of the anterior cruciate ligament (ACL) remain a persistent health issue across a range of multi-directional team sports such as soccer, basketball, or handball (1). Such ACL injuries typically occur during jump-landing and/or change-of-direction (COD) maneuvers with no or minimal physical contact with an opponent (2–4). This observation has inspired many research groups to develop injury prevention exercise programs (IPEPs) aimed at reducing the risk of non-contact ACL injuries in multi-directional team sports (5–8). The respective training programs usually include generic strength and balance exercises combined with jump-landing and COD technique training to modify high-risk movement and associated mechanical loading (9). The classical ACL injury-prone pattern addressed by IPEPs is a landing posture characterized by knee extension, knee valgus, and trunk lateral bending over the stance leg (i.e., opposite to intended movement direction), which is associated with high tibial compressive forces and knee abduction moments—both linked to the ACL injury mechanism (10–12). While some IPEPs have shown promising efficacy in reducing the risk of knee injury in randomized-controlled trials, the effectiveness of such programs in realworld settings has been found to be much lower (13) while ACL injury incidences continue to rise (14, 15).

Although the underlying reasons for this gap in ACL injury prevention are likely multifactorial, current evidence-based IPEPs have been criticized for not reflecting the state-of-the-art in motor learning and motor control research (16–18). For example, three common IPEPs [FIFA11+, Knee Control, and Prevent Injury and Enhance Performance (PEP)] rely on jump-landing and COD drills that stem from an arguably narrow biomechanical perspective on ACL injury prevention, i.e., they focus on pre-planned movement tasks and prescriptive technique instructions simply aimed at avoiding “high-risk” biomechanical patterns (18). This neglects an ecological dynamics perspective on human movement and motor learning, which views movement as an emergent result of three types of constraints; organism (athlete) constraints, environmental constraints and task constraints (19). Following this view, ACL injury prevention training should try to manipulate environmental and task constraints within sport-specific boundaries in a way that allows the athlete's motor control system to learn and explore optimal movement patterns in a self-organizing manner (20).

Such a constraints-led training approach has shown promising effects in a previous study, which investigated improvements in 45° COD movement patterns after a 12-week COD training program following a “non-linear pedagogy” (21), which purposefully manipulated constraints to foster COD execution variability (18). The actual training content, training adherence, and baseline performances, however, were not reported in the previous study. Furthermore, since the previous study standardized the COD approach speed between baseline and follow-up testing without quantifying other aspects of COD performance, it is unclear whether a constraints-led training approach can overcome the well-known performance-injury conflict in COD maneuvers (22, 23). Specifically, it is unknown whether a training approach that fosters COD execution variability with minimal technique feedback can both reduce risk factors for ACL injuries during COD while improving or at least maintaining COD performance.

We explored a one-on-one laser tag game as a constraints-led training method to improve COD movement patterns. We hypothesized that this game would improve COD performance and reduce COD injury risk factors, specifically the peak knee abduction moment (pKAM) and trunk lateral bending, for the following reasons. First, the game inherently elicits many repetitions of rapid CODs as players repeatedly evade an opponent's laser, promoting practice-driven refinement of COD movement strategies. Second, successful evasion requires players to execute unpredictable CODs, fostering variability in COD execution angle and speed that can guide self-organization toward more efficient and faster movement solutions. Third, the simultaneous aiming and evasion tasks encourage exploration of an optimal trade-off between highly unpredictable movements and sufficiently stable patterns that still permit accurate aiming. Fourth, because the targets are worn on the upper body, players must actively experiment with lateral trunk movements and axial trunk rotations during CODs; given the strong association between trunk lateral bending and pKAM (12), this exploration may bias learning toward trunk strategies that lower frontal-plane knee loading. Taken together, these constraints-led features of one-on-one laser tag provided the rationale for our hypotheses that training would reduce pKAM and trunk lateral bending while maintaining or improving COD performance.

The primary aim of this study was to investigate the hypothesis (H1) that an eight-week laser tag training intervention leads to a reduction in the pKAM during a 135° COD with a (H2) concurrent increase in COD performance quantified by the COD completion time, angle, and ground contact time. The secondary aim was to test the hypothesis (H3) that laser tag training leads to reductions in peak trunk lateral bending during the 135° COD as a potential mechanism for reduced pKAMs. Effects of laser tag training were compared to training effects observed in a previous study of a similar study design but with a training focus on more traditional neuromuscular conditioning and either COD technique training or linear sprint training (9).

## 2 Methods

### 2.1 Study design and participants

This study was a follow-up experiment to a previous training intervention study aimed at limiting movement patterns associated with ACL injury risk during COD (9). The previous study compared the effects of an 8-week neuromuscular training program in a group of highly active sport science students with either a 1) COD technique training focus (CODG) or a 2) linear sprint training focus (CG). The CG served as a control group with a similar training intensity to the CODG but without any improvements in COD technique. For the current study, we selected the required sample size of *n* = 12 for the laser training group (LASG) in analogy to the sample size estimation in the previous study (9). Participants in the current follow-up study included *n* = 12 highly active sport science students (75% female) who met the following inclusion criteria: (1) sport science student at the University of Innsbruck, (2) 18–40 years old, (3) experience with multi-directional sports that involve CODs, and (4) an absence of lower extremity injuries in the last six months prior to entry into the study. This study was approved by the local ethics committee at the Department of Sport Science at the Universität Innsbruck (Board for Ethical Issues, Review Board Sport Science, ID 68/2,022) following guidelines on ethical principles for research involving human subjects as outlines in the Declaration of Helsinki. All participants provided written informed consent before starting the training or measurements. A preliminary analysis of this data set has been published in abstract form (24).

### 2.2 Training intervention

**Figure 1:**
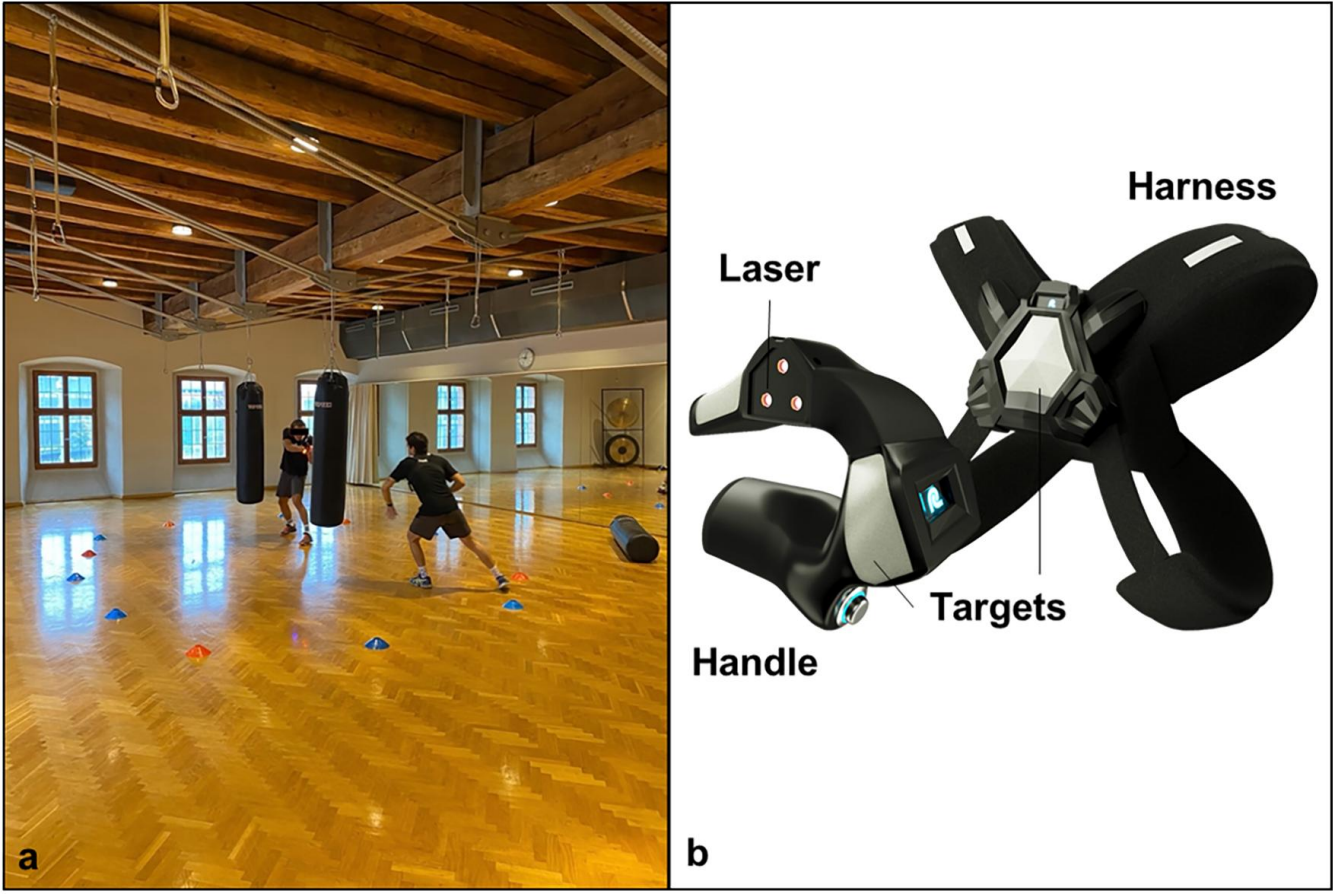
Example for open laser tag game with added punching bags as obstacles **(a)** and image of the laser tag handle and harness **(b)**.

All participants were asked to complete at least two 30-minute laser tag training sessions per week over eight weeks. The laser tag system was provided by the company LAZR Fitness (Lazeur SA, Geneva, Switzerland) and included four laser tag devices consisting of a handle to aim the laser and a chest harness to detect the laser beams from other players. In addition, the laser tag system included a central server for registering players with their handles and harnesses and for counting points during laser tag exercises. Importantly, given the low energy of the lasers used (class 2 lasers, power < 1 mW), there was no risk of eye or skin damage associated with the laser beam-emitting handles. All training sessions were supervised by a sport scientist with additional experience as an athletic trainer (co-author FS). Most training sessions took place in two different indoor gyms with either wooden or linoleum flooring while some selected training sessions took place outdoors on an artificial turf field. This variation in surface characteristics was purposefully selected to create additional training stimuli in response to varying environmental constraints. All training sessions were limited to a maximum of four participants given the number of laser tag handles available. Each training session followed a fixed structure that was kept constant over the eight weeks: The first part was a generic warm-up consisting of low-intensity running, mobility, and COD exercises similar to the first six warm-up exercises of the FIFA11 + program (25). The second part was a laser tag specific warm-up, which consisted of side-shuffle and COD movements while trying to aim the laser at a stationary harness (2 × 20 s with 30 s break). The third part were partner laser tag exercises, which consisted of one aiming player trying to aim the handle at a second moving player trying to evade the laser (4 × 20 s with 30 s break). For these exercises, the evading player had to move according to spatial constraints that were varied throughout the eight weeks of training. In the first two weeks, the evading player was shuffling and running around a circle while the aiming player was moving within the circle. In the third and fourth weeks, the evading player shuffled back and forth on a straight line performing CODs to evade the laser of the aiming player. In the fifth and sixth week, the evading player had to move inside a square while the aiming player could move around the outside of the square. In the final two weeks, the evading player moved within a triangle while wearing an additional harness on the back, which enabled a second target for the aiming player moving around the outside of the triangle. These variations in spatial and task constraints were selected to evoke a wide range of COD movement strategies that the evading players had to explore to minimize the number of hits by the aiming player. The final part of the training sessions was an open laser tag game (4 × 20 s with 10 s break) where players moved freely within a constrained space trying to hit the other players while avoiding being hit themselves. During the eight weeks the specified space was slightly varied, e.g., by adding obstacles inside the space (see Figure 1), to encourage variable playing strategies and COD movement patterns. In addition, the exercise durations and number of exercise repetitions was progressively increased throughout the eight weeks to also challenge the participants’ cardiovascular system next to the motor control system. A detailed description of the training program can be found in Supplementary Table 1. The details of the training program within the CODG and CG can be found in the initial publication (9).

Most importantly, the participants did not receive any technique instructions during any of the training sessions and thus were free to complete COD and shuffle movements using their preferred strategy.

### 2.3 Experimental procedures

The experimental protocol and data collection were conducted in analogy to the procedures described in the previous COD training study (9). To summarize, baseline and follow-up measurements were carried out in a biomechanics laboratory within 1–2 weeks before and after the 8-week training program. Participants were equipped with a 56-marker full-body reflective marker set, which was an extended version of the Vicon Plug-in Gait full body marker set. Following a standardized warm-up on a stationary bike, all participants completed submaximal 45° and 135° CODs for a specific warmup and for familiarization with the investigated 135° COD task. The familiarization ended when participants could demonstrate three successful 135° CODs that met the following criteria: (1) penultimate left foot contact on a first ground-embedded force plate, (2) final right foot contact on an adjacent second force plate, (3) pronounced COD step on the second force plate to complete the 135° COD while avoiding a rounded turn, and (4) perform the COD task at maximum speed. The force plates were covered with a criss-cross pattern of athletic tape to reduce the risk of slipping. Timing gates were placed at a 5 m distance to the center of the second force plate marking the start and at a 2.7 m distance from the second force plate marking the finish line.

During the actual data collection, participants performed six successful preplanned 135° CODs at a maximum speed with at least one minute of rest in between trials. The trajectories of the reflective markers were captured by a 10-camera motion capture system (Vicon, Oxford, UK) with a sample rate of 250 Hz while ground reaction forces were captured by one Kistler plate for the penultimate contact (Kistler, Winterthur, Switzerland) and one AMTI plate for the final foot contact (AMTI, Watertown, MA, USA) both sampling at 1,000 Hz. The COD completion time was provided by the timing gates (Brower Timing Systems, Draper, UT, USA). In addition to the dynamic trials, static standing trials in a “T-Pose” were recorded for all participants.

### 2.4 Data processing

All data processing steps were analogous to the procedures described previously (9). Briefly, all marker trajectories were reconstructed within Vicon Nexus software (v. 2.14, Vicon, Oxford, UK).

All marker trajectories and force data were filtered using a third-order dual-pass low-pass Butterworth filter with a cut-off frequency at 15 Hz. Biomechanical modeling of the COD movements was then conducted in OpenSim software (v. 4.2) (26) while using the whole-body musculoskeletal model of Catelli and colleagues (27) with two added degrees of freedom at the knee, i.e., knee adduction-abduction (constrained to −10° to +10°) and knee internal-external rotation (constrained to −40° to +40°). The knee joint rotation sequence was (1) flexion, (2) adduction-abduction, (3) internal-external rotation. The procedures in OpenSim included model scaling, inverse kinematics to obtain joint angles as well as the center-of-mass trajectory, and inverse dynamics to obtain joint moments. Based on the outputs of the OpenSim software, the following outcome variables were determined in a custom-written MATLAB script (The MathWorks Inc., Natick, MA, USA): (i) the pKAM within the first 25% of the final foot contact, (ii) the peak lateral trunk lean during the final foot contact, (iii) the ground contact time of the final foot contact, (iv) the executed COD angle based on the angle of the vector of the horizontal COM velocity when entering the COD compared to the same vector when exiting the COD, and (v) the approach speed based on the COM speed in the horizontal plane at the initial contact of the penultimate foot contact.

### 2.5 Data analysis and statistics

As described in detail by Mohr and colleagues (9), we used linear mixed effects models to investigate the fixed effects of the factors “training” (baseline vs. follow-up) and “group” (CODG, LASG, CG) and their interaction (“training x group”) on the outcome variables described above. All models included a random intercept for “participant” to model the variability in outcome variables among six COD repetitions per participant as well as variable baseline values across participants (28). Further, the mixed effects models related to the biomechanical outcome variables “pKAM” (hypothesis H1) and “peak lateral trunk lean” (hypothesis H3) were adjusted for the covariates “sex”, “approach speed”, and “COD executed angle” to avoid potential confounding by these variables. The mixed effects models related to the performance outcome variables “ground contact time”, “executed COD angle”, “approach speed”, and “COD completion time” (hypothesis H2) were only adjusted by the covariate “sex”. The model assumptions for linear mixed models (29) were assessed and confirmed based on the following procedures: (1) normal distribution of residuals based on Q-Q plots and Kolmogorov–Smirnov tests of residuals, (2) constant variance of residuals (i.e., homoscedasticity) based on scatter plots of predicted vs. residual values, and (3) normal distribution of random effects based on random coefficients histograms. Interaction and main effects were evaluated based on the *F*-tests associated with the fixed effects, which can be interpreted analogous to a classic analysis of variance table (30). In the presence of significant “training” or “training x group” effects, a simple effects analysis was carried out to investigate significant training effects specific to each training group. We rejected our null hypotheses if there were significant “training x group” interactions and when the simple effects analyses indicated significant and favorable training effects within the LASG group. Cohen's d effect sizes were determined from the t-statistic of the simple effects analysis following the recommendations by Lakens (31) and interpreted according to guidelines by Cohen (32).

All statistical analyses were conducted in jamovi software (33) using the GAMLj module (v. 2.6.6) (30) with its default settings (simple factors coding and centered covariates). The significance level was set to alpha = 0.05.

## 3 Results

Participant characteristics and training adherence within each training group can be found in Table 1. The average training adherence was the highest in LASG (mean ± SD, 2.0 ± 0.2 sessions/week) followed by the CG (1.7 ± 0.4 sessions/week) and the CODG (1.4 ± 0.3 sessions/week).

**Table 1:** Participant characteristics.

Variable*	LASG *n* = 12	CODG *n* = 11	SG *n* = 11
Proportion of female participants [%]	75	36	45
Age [years]	24.3 ± 2.4	24.3 ± 2.0	22.3 ± 1.6
Height [cm]	173 ± 10	176 ± 10	174 ± 12
Body mass [kg]	69 ± 11	71 ± 8	66 ± 9
Frequency of training sessions per week	2.0 ± 0.2	1.4 ± 0.3	1.7 ± 0.4

* All variables except “Proportion of female participants” show the mean ± standard deviation.

### 3.1 Peak knee abduction moment

**Figure 2:**
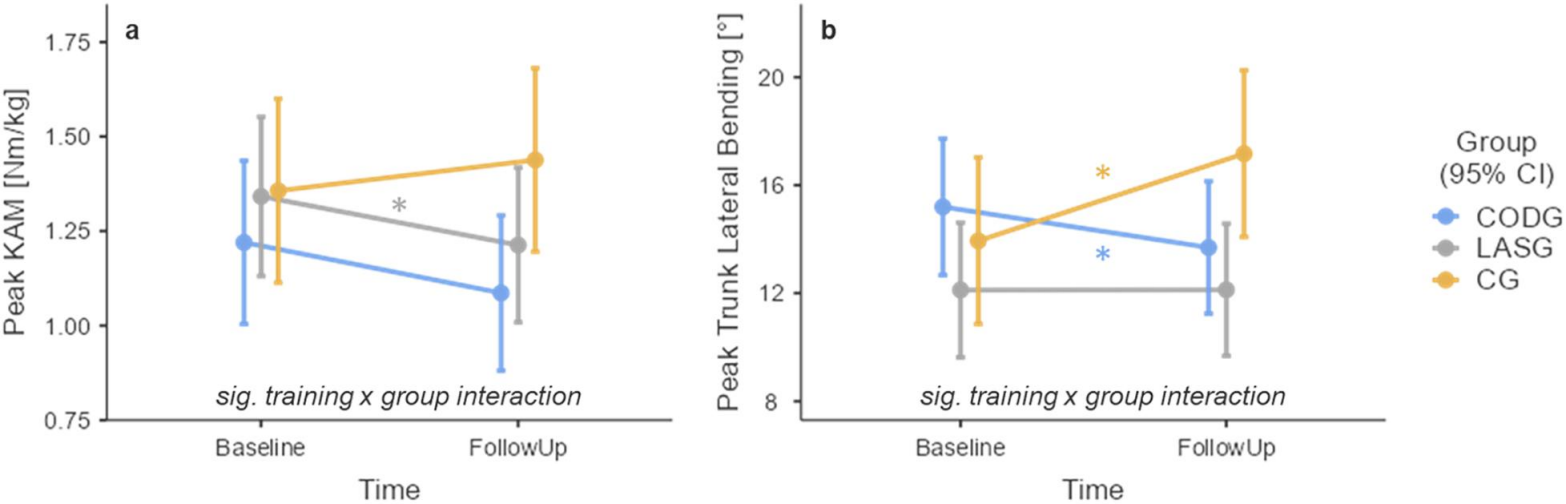
Training effects on the pKAM **(a)** and peak trunk trunk lateral bending angle **(b)** each panel shows the respective estimated marginal means and 95% confidence intervals (CI) of the COD technique training (CODG, blue), laser tag training (LASG, grey), and the linear sprint training (CG, orange). Asterisks mark statistically significant changes from baseline to follow-up. Please note that these means are adjusted for the factor sex and covariates COD approach speed and COD angle.

There was a significant “training x group” interaction effect [F(2,315.6) = 3.641, *p* = 0.027] with respect to the pKAM (Figure 2a). The simple effects analysis showed a statistically significant reduction of the pKAM from baseline to follow-up in the LASG with a moderate effect size [mean [95% CI] of follow-up minus baseline: −0.13 [−0.25, −0.00] Nm/kg, *p* = 0.038, d = 0.63]. The CODG showed a similar reduction in the pKAM [−0.13 (−0.27, 0.00) Nm/kg, *p* = 0.056, d = 0.58] while the CG showed a slight increase in the pKAM [0.08 (−0.05, 0.21) Nm/kg, *p* = 0.222, d = 0.37].

### 3.2 COD performance

**Figure 3:**
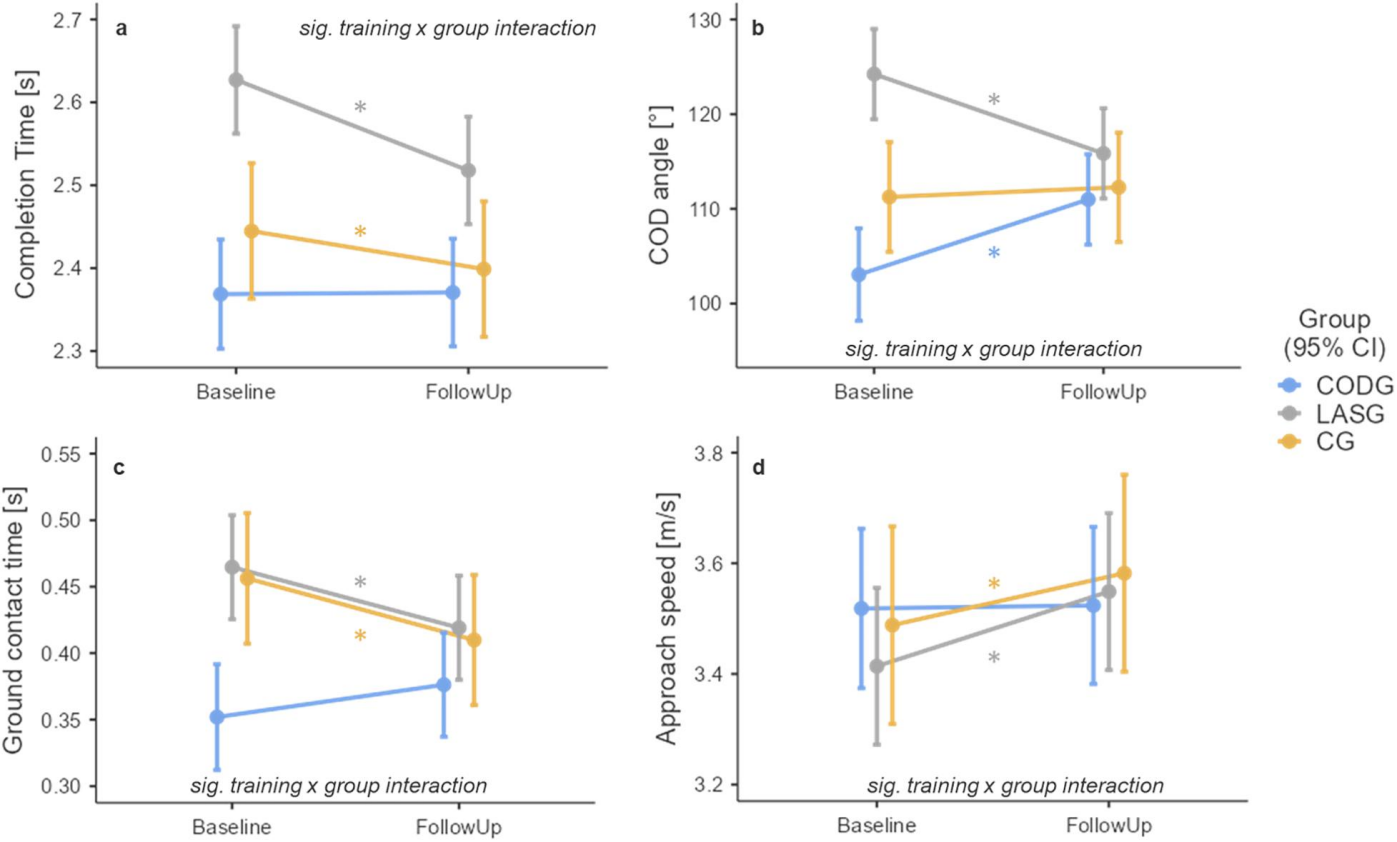
Training effects on the COD completion time **(a)**, COD angle **(b)**, ground contact time **(c)**, and approach speed **(d)** each panel shows the respective estimated marginal means and 95% confidence intervals (CI) of the COD technique training (CODG, blue), laser tag training (LASG, grey), and the linear sprint training (CG, orange). Asterisks mark statistically significant changes from baseline to follow-up. Please note that these means are adjusted for the factor sex.

There was a significant “training x group” interaction effect [F(2,313) = 14.930, *p* < 0.001] with respect to the COD completion time (Figure 3a). The simple effects analysis showed a statistically significant reduction of the completion time from baseline to follow-up in the LASG with a large effect size [−0.11 (−0.14, −0.08) s, *p* < 0.001, d = 2.47]. The CG also showed a reduction in completion times [−0.05 (−0.08, 0.01) s, *p* = 0.004, d = 0.88] while the CODG showed no average change [0.00 (−0.03, 0.03) s, *p* = 0.898, d = 0.04].

There was a significant “training x group” interaction effect [F(2,313.6) = 41.384, *p* < 0.001] with respect to the COD executed angle (Figure 3b). The simple effects analysis showed a statistically significant reduction of the executed angle from baseline to follow-up in the LASG with a large effect size [−8.4 (−10.7, −6.1)°, *p* < 0.001, d = 2.15]. The CODG showed an increased COD executed angle at follow-up [7.9 (5.2, 10.7)°, *p* < 0.001, d = 1.72] while the CG showed a minimal average change [1.0 (−1.7, 3.7)°, *p* = 0.466, d = 0.22].

There was a significant “training x group” interaction effect [F(2,311.2) = 17.81, *p* < 0.001] with respect to the ground contact time of the COD final step (Figure 3c). The simple effects analysis showed a statistically significant reduction of the ground contact time from baseline to follow-up in the LASG with a large effect size [−0.05 (−0.06, −0.03) s, *p* < 0.001, d = 1.65]. The CG showed a comparable reduction in ground contact time [−0.05 (−0.07, 0.03) s, *p* < 0.001, d = 1.42] while the CODG showed an increased time [0.02 (0.00, 0.04) s, *p* = 0.014, d = 0.74].

There was a significant “training x group” interaction effect [F(2,314.5) = 4.090, *p* = 0.018] with respect to the COD approach speed (Figure 3d). The simple effects analysis showed a statistically significant increase in approach speed from baseline to follow-up in the LASG with a large effect size [0.13 (0.07, 0.19) m/s, *p* < 0.001, d = 1.38]. A similar increase in approach speed was observed in the CG [0.10 (0.03, 0.16) m/s, *p* = 0.007, d = 0.82] while the CODG showed no average change in approach speed [0.00 (−0.06, 0.07) m/s, *p* = 0.875, d = 0.05].

### 3.3 Peak trunk lateral bending

There was a significant “training x group” interaction effect [F(2,315.2) = 17.003, *p* < 0.001] with respect to the peak lateral trunk bending angle (Figure 2b). The simple effects analysis showed no statistically significant changes in peak lateral trunk bending from baseline to follow-up in the LASG and a negligible effect size [0.01 (−1.08, 1.09)°, *p* = 0.990, d = 0.01]. The CODG showed reduced peak lateral trunk bending at follow-up [−1.50 (−2.72, −0.29)°, *p* = 0.015, d = 0.74] while the CG showed an increased angle at follow-up [3.23 (2.06, 4.39)°, *p* < 0.001, d = 1.65].

## 4 Discussion

This study investigated whether an eight-week laser tag training as a constraints-led training method for COD movements can improve movement technique in the context of ACL injury risk and performance during a maximum-speed 135° COD. The findings support our first and second hypotheses, that the laser tag training reduced the pKAM as a risk factor for ACL injuries and improved COD performance in terms of COD speed and ground contact time. Our third hypothesis that the reduction in the pKAM can be explained by a training-induced reduction in peak trunk lateral bending was not supported by the results of this study.

The 10% reduction in the pKAM following laser tag training was of similar magnitude compared to the COD technique modification in the previous study (9) demonstrating that a constraints-led training method without technique instructions can achieve comparable reductions in COD frontal plane knee loading. This is in alignment with previous reports (21, 34) showing a benefit of training methods that induce variability in movement execution and movement strategy in order to reduce biomechanical risk factors for ACL injury, e.g., the pKAM, during COD and landing tasks. However, these studies only loosely standardized performance [COD approach speed between 4.5–5.5 m/s (21)] or did not report on performance (34). In consequence, it is unknown whether the improved and safer movement technique following training came at the expense of slower CODs or lower jump height, a phenomenon known as the performance-injury conflict (22, 23, 35). In the current study, the laser tag training group showed reduced pKAMs and in parallel improved their COD performance as evidenced by reduced completion times, i.e., faster COD speeds, and reduced ground contact times. These training effects are in contrast to the COD technique training group, which showed reductions in the pKAM with performance decrements and in contrast to the linear sprint training group, which showed improved performance but slightly higher pKAMs following training (9). In consequence, the constraints-led laser tag training investigated in the current study may help to overcome the performance-injury conflict, which is a strong argument for coaches to actually implement such training programs in their practice (36).

One limiting factor for the above statement is the reduction in COD executed angle observed at the follow-up test in the laser tag training group, which could have acted as a confounder given the known association between COD angle and pKAM (35). However, this association has only been observed between 30° to 90° but not for COD angles beyond 90° (37) as present in the current study. In addition, since the COD executed angle was incorporated as a covariate in our statistical model of pKAM, it is unlikely that the finding of reduced pKAMs in the laser tag training group was due to more shallow COD angles at follow-up testing. The lower COD executed angle could, however, help explain the reduced ground contact and COD completion times observed in the laser tag group post-training because of lower impulses required to change momentum for more shallow COD angles (35).

The absence of systematic changes in trunk lateral bending before and after laser tag training contradicts our third hypothesis that a reduction in peak trunk lateral bending could have served as a mechanism to achieve lower pKAMs post-training. This hypothesis assumed that the rapid and variable trunk motions required by the laser tag game would guide the athletes towards efficient trunk movement patterns that enhance performance but also reduce joint loading. Reduced trunk lateral bending opposite to the intended movement direction is associated with lower pKAM (38, 39) and improved performance (40) and thus we expected the laser tag participants to self-optimize towards this movement pattern. Our findings, however, do not support such a mechanism, which begs the question how the laser tag training led to systematically reduced pKAMs. Our current working hypothesis is that the highly variable COD movement patterns required by the laser tag game led to athlete-individual adaptations in COD kinematics that were generally beneficial for reducing the pKAM. In fact, four out of eleven athletes of the laser tag group did show a reduction in peak trunk lateral bending of at least 2.5° at the follow-up test while the other athletes showed minimal changes or an increased angle. Other athletes may have achieved a lower pKAM through a reduced knee abduction angle or hip internal rotation angle as observed for the COD technique training group in our previous study (9). Such athlete-individual kinematic adaptations at different joints would not become evident in a statistical analysis at the group level. Athlete-individual kinematic adaptations to laser tag training also seem reasonable when one considers that each athlete is constrained by his or her motor capacity, e.g., determined by muscle strength and flexibility, which put athlete-individual bounds on the emergence of new movement patterns during training (18). With only two testing sessions and six COD repetitions per session, however, our study design could not reliably investigate athlete-individual training adaptations. Future studies should incorporate a higher number of COD repetitions per session and include more frequent testing sessions, e.g., one per week, to clearly track athlete-individual evolution in COD kinematics and kinetics throughout a constraints-led or traditional technique modification training.

### 4.1 Conclusion

Eight weeks of constraints-led laser tag training aimed at improving COD movement strategies led to reductions in the pKAM and improved performance in terms of COD speed and ground contact time, however, at the cost of more shallow COD executed angles during maximum-speed 135° COD movements. As such, constraints-led training approaches may be useful to overcome the performance-injury conflict when training to reduce large pKAMs in COD movements for ACL injury prevention.

## Data Availability

The raw data supporting the conclusions of this article will be made available by the authors, without undue reservation.
