# Supplementary material for: Laser tag training reduces knee abduction moments and improves performance during change-of-direction movements
Source: Front Sports Act Living. 2026 Feb 23;8:1686129. doi: 10.3389/fspor.2026.1686129 (PMC12968199; doi:10.3389/fspor.2026.1686129)
Supplement: Supplementary file 1 [file Datasheet1.pdf]

These plans are related to weeks W1 - W4 of the training intervention

W1 + W2			
**Exercise**	**Repetitions**	**Instructions**	**Time**
Generic warm-up			
Running	4 x 30 m, back and forth	Slow to moderate speed, slightly faster on the way back	5-8 min
Lateral Skips R/L	1 x 20 m each side, run back	Moderate speed	
Lateral Hops R/L	1 x 20 m each side, run back		
Lateral Skips w/ diagonal posterior knee R/L	1 x 20 m each side, run back		
Lateral Hops w/ diagonal posterior knee R/L	1 x 20 m each side, run back		
Diagonal Skips	2 x 20 m each side, run back		
Diagonal Hops	2 x 20 m each side, run back		
Incremental set Runs	2 x 30 m, run back	Increasing speed up to 80% of max speed	
Laser tag specific warm-up			
Side Shuffle + 180° COD while aiming at stationary harness	2 x 20 sec, 30 sec rest	Keep the laser on the target while shuffling to the side	3 min
Laser tag partner exercises			
Aim & Evade<br>Spatial Constraint: Circle	Week 1: 4 x 20 sec, Week 2: 4 x 30 sec<br>30 sec rest to switch roles with the partner	Evading player tries to avoid beeing hit by the partner by constantly moving outside the circle.<br>The aiming player tries to hit the evading players' target while shuffling inside the circle.	7 min
Laser tag game			
Open laser tag game	4 x 20 sec game, 10 sec rest	All players try to hit the opponents' target while evading the opponents' laser.	7 min

W3 + W4			
**Exercise**	**Repetitions**	**Instructions**	**Time**
Generic warm-up			
Running	4 x 30 m, back and forth	Slow to moderate speed, slightly faster on the way back	5-8 min
Lateral Skips R/L	1 x 20 m each side, run back	Moderate speed	
Lateral Hops R/L	1 x 20 m each side, run back		
Lateral Skips w/ diagonal posterior knee R/L	1 x 20 m each side, run back		
Lateral Hops w/ diagonal posterior knee R/L	1 x 20 m each side, run back		
Diagonal Skips	2 x 20 m each side, run back		
Diagonal Hops	2 x 20 m each side, run back		
Incremental set Runs	2 x 30 m, run back	Increasing speed up to 80% of max speed	
Laser tag specific warm-up			
Side Shuffle + 180° COD while aiming at stationary harness	2 x 20 sec, 30 sec rest	Keep the laser on the target while shuffling to the side	3 min
Laser tag partner exercises			
Aim & Evade<br>Spatial Constraint: Separating line	Week 3: 4 x 20 sec, Week 4: 4 x 30 sec<br>30 sec rest to switch roles with the partner	Aiming and evading players try to hit and evade each other while constantly shuffling and side-cutting along the separating line.	7 min
Laser tag game			
Open laser tag game	6 x 20 sec game, 10 sec rest	All players try to hit the opponents' target while evading the opponents' laser.	8 min

These plans are related to weeks W5 - W8 of the training intervention

W5 + W6			
**Exercise**	**Repetitions**	**Instructions**	**Time**
Generic warm-up			
Running	4 x 30 m, back and forth	Slow to moderate speed, slightly faster on the way back	5-8 min
Lateral Skips R/L	1 x 20 m each side, run back	Moderate speed	
Lateral Hops R/L	1 x 20 m each side, run back		
Lateral Skips w/ diagonal posterior knee R/L	1 x 20 m each side, run back		
Lateral Hops w/ diagonal posterior knee R/L	1 x 20 m each side, run back		
Diagonal Skips	2 x 20 m each side, run back		
Diagonal Hops	2 x 20 m each side, run back		
Incremental set Runs	2 x 30 m, run back	Increasing speed up to 80% of max speed	
Laser tag specific warm-up			
Side Shuffle + 180° COD while aiming at stationary harness	2 x 20 sec, 30 sec rest	Keep the laser on the target while shuffling to the side	3 min
Laser tag partner exercises			
Aim & Evade<br>Spatial Constraint: Square	Week 5: 4 x 20 sec, Week 6: 4 x 30 sec<br>30 sec rest to switch roles with the partner	The evading player tries to avoid beeing hit by the partner by constantly moving inside the square.<br>The aiming player tries to hit the target while shuffling outside of the square.<br>Utilized variation: On two sides of the square, the evading player can only perform forward/backward motions while on the other two, the player can only perform side-shuffle and cut movements.	7 min
Laser tag game			
Open laser tag game	8 x 20 sec game, 10 sec rest	All players try to hit the opponents' target while evading the opponents' laser.	9 min

W7 + W8			
**Exercise**	**Repetitions**	**Instructions**	**Time**
Generic warm-up			
Running	4 x 30 m, back and forth	Slow to moderate speed, slightly faster on the way back	5-8 min
Lateral Skips R/L	1 x 20 m each side, run back	Moderate speed	
Lateral Hops R/L	1 x 20 m each side, run back		
Lateral Skips w/ diagonal posterior knee R/L	1 x 20 m each side, run back		
Lateral Hops w/ diagonal posterior knee R/L	1 x 20 m each side, run back		
Diagonal Skips	2 x 20 m each side, run back		
Diagonal Hops	2 x 20 m each side, run back		
Incremental set Runs	2 x 30 m, run back	Increasing speed up to 80% of max speed	
Laser tag specific warm-up			
Side Shuffle + 180° COD while aiming at stationary harness	2 x 20 sec, 30 sec rest	Keep the laser on the target while shuffling to the side	3 min
Laser tag partner exercises			
Aim & Evade<br>Spatial Constraint: Triangle	Week 7: 4 x 20 sec, Week 8: 4 x 30 sec<br>30 sec rest to switch roles with the partner	The evading player tries to avoid beeing hit by the partner by constantly moving inside the triangle.<br>The aiming player tries to hit the target while shuffling outside of the triangle.<br>Utilized variation: The evading player is wearing an additional target on his/her back to increase the difficulty.	7 min
Laser tag game			
Open laser tag game	10 x 20 sec game, 10 sec rest	All players try to hit the opponents' target while evading the opponents' laser.<br>Variations with added obstacles within the playing area, e.g. punching bags and gym boxes.	10 min
